# Supplementary material for: Rapid environmental change in games: complications and counter-intuitive outcomes
Source: Sci Rep. 2019 May 14;9:7373. doi: 10.1038/s41598-019-43770-x (PMC6517380; doi:10.1038/s41598-019-43770-x)
Supplement: Supplementary file 1 — Supplementary Information [file 41598_2019_43770_MOESM1_ESM.docx]

**Rapid environmental change in games:**

**complications and counter-intuitive outcomes**

Pete C Trimmer*, Brendan J Barrett, Richard McElreath, Andrew Sih

* Corresponding author: pete.trimmer@gmail.com

**Supplementary Information 1: HIREC Calculations**

*First, re-capping the equations and values of the main text*

Denoting the probability of an individual playing hawk by *h*, then prior to HIREC, the expected payoff of playing hawk is: (1 – *h*)*V* + *h*(*V* – *C*)/2, and the expected payoff of playing dove is: (1 – *h*)*V*/2. Setting these equal, we find the optimal probability of playing hawk, *h* = *V*/*C*.

Assuming that, before HIREC, the reward for an uncontested resource was *V* = 1, and the cost of a contest was *C* = 2, then *h* = ½, with an expected payoff (to any individual) of (1 – *h*)*V*/2 = 0.5*1/2 = 0.25.

*Consequences of mechanisms which determine phenotype post HIREC*

We now consider the effect of HIREC decreasing the value of rewards, say from *V* = 1 to 0.5. What will be the effect?

If the behavioral differences were the result of genetic polymorphisms

Immediately after HIREC, the hawks would get a payoff following HIREC, of:

(1 – *h*)*V* + *h*(*V* – *C*)/2 = (1 – 0.5)*0.5 + 0.5*(0.5 – 2)/2 = -0.125.

Thus, on average, the hawks would leave the scenario with less energy than that with which they entered the scenario.

The doves would still get a positive payoff, of:

(1 – *h*)*V*/2 = (1 – 0.5)0.5/2 = 0.125.

Consequently, only the doves would, on average, gain reproductive value in interactions.

If the behavioral differences were the result of probabilistic choice on each encounter

If, instead, the same balance (of *h* = 0.5) had ancestrally come about by each individual randomizing their choice in each encounter then, following HIREC, each individual would get the same mean payoff, as the average of the return to hawks and doves above; thus their expected benefit would be the mean of -0.125 (when behaving as a hawk, which occurs 50% of the time) and +0.125 (when behaving as a dove, on the other 50% of occasions); i.e., an expected value of 0. (Note that this is looking at choices immediately after HIREC, before *h* is able to alter.)

If the behavioral differences were the result of an evolved reaction-norm in relation to the value of food

The third possibility is that individuals may have evolved in circumstances where their likelihood of acting as hawk had evolved in relation to the strength of food cue that they received (i.e., letting *h* for each encounter depend on the value *V*). Under these circumstances, the individuals would display a reaction norm to the food rewards, increasing the probability of playing hawk as the perceived reward increased. Under such circumstances, they would still achieve the same expected payoff pre-HIREC (of 0.25) but, following HIREC, they would immediately adopt the best possible behavior, which continues to be given by *h* = *V*/*C*.; i.e., playing hawk on 25% of occasions when *V* = 0.5 and *C* = 2.

This results in a positive expected payoff of (1 – *h*)*V*/2 = (1 – .25)0.5/2 = 0.1875, to both hawks and doves. Thus, although the rate of reproductive success would have decreased due to HIREC, each member of the population would still tend to accrue reproductive value with numerous interactions – and would do even better than the doves in the genetic polymorphism case.

General cases for the main text

*Genetic polymorphisms:*

Expected payoff to hawks is (1 – *h*)*V* + *h*(*V* – *C*)/2.

Expected payoff to doves is (1 – *h*)*V*/2.

With *h* = 0.5 (as we assume has evolved with the initial values of *V* = 1, *C* = 2), these payoffs become: 3*V*/4 – *C*/4 for hawks, and *V*/4 for doves.

*Probabilistic choice:*

The expected payoff is the mean of the payoff to hawks and doves above; i.e., *V*/2 – *C*/8.

*Optimal reaction norm:*

Having updated the probability of playing hawk to *V*/*C*, the expected payoff to individuals is then (1 – *V*/*C*)*V*/2.

*Matlab code for Figure 1:*

c = 2; oldv = 1; h = oldv/c; v = [0.01:0.01:2];

y1 = (1-v/c).*v/2; % Reaction norm

y3 = (1.0-h)*v + h*(v-c)/2; % Genetic hawks

y4 = (1.0-h)*v/2; % Genetic doves

y2 = h*y3 + (1-h)*y4; % Probabilistic choice

plot(v,y1); hold on; plot(v,y2); hold on; plot(v,y3); hold on; plot(v,y4)

*Matlab code for Figure 2:*

oldc = 2; v = 1.0; h = v/oldc; c = [1:0.01:3];

y1 = (1-v./c)*v/2; % Reaction norm

y3 = (1.0-h)*v + h*(v-c)/2; % Genetic hawks

y4(1:201) = (1.0-h)*v/2; % Genetic doves

y2 = h*y3 + (1-h)*y4; % Probabilistic choice

plot(c,y1); hold on; plot(c,y2); hold on; plot(c,y3); hold on; plot(c,y4)

**Supplementary Information 2: Other Games**

The Producer-Scrounger Game

Numerous situations involve ‘producers’ and ‘scroungers’. For instance, to acquire food, an individual can search for new food sources (thus producing new options – and information – largely independently of what others are doing) or seek-out other individuals who have found a food source, and scrounge some of that food. Barnard & Sibly (1981) identify that, for a particular number of individuals, natural selection will tend to result in a particular balance of producers to scroungers (an ESS).

Just as with the hawk-dove game, the same frequency balance could be struck (approximately) in numerous different ways; a proportion of individuals being born of each type (genetic), actions being learned in relation to the rewards from trying each option (ontogenetic), or cues from food (or from producers when food is found) may govern the extent to which individuals switch from producing to scrounging behavior. Each of these can result in a similar overall ratio of scroungers to producers (which is optimal pre-HIREC), but the mechanism causing that balance will result in very different outcomes following HIREC.

- In the case of genetic differences, one of the strategies (i.e., either producers or scroungers) are likely to do less well than the other strategies following HIREC. The rate at which the ratio of producers to scroungers alters will depend on the selection pressures (if selection is weak, the change in ratio may be very slow).
- If each individual in the system is learning about the benefit of producing to scrounging behavior during their development then, following HIREC, the new balance may be struck within a generation.
- If individuals are actually sensing the amount of food available under each option (cf. matching rule), then the transition to the new optimum following HIREC may be almost instantaneous.
- There are other possibilities, of course. For instance, individuals may be able to sense the amount of food available at existing scrounging sites, but have little knowledge about the amount of food they would obtain from foraging sites, instead going by evolved ‘traits’ of what to settle for at scrounging sites.

We model each of these in turn to show how HIREC can lead to different predictions, depending on the underlying scenario. There are many versions of producer-scrounger games, with different levels of information sharing. We base our analysis on the scenario described by Giraldeau & Beauchamp (1999), which we now summarise.

The scenario is assumed to involve *G* foragers, of which, at equilibrium, a proportion of the population, $q$, are producers while $\left( 1-q \right)$ are scroungers. Producers encounter food at a known rate, λ. Each piece of food contains *F* units; at the point of discovery, the producer immediately obtains *a* (0 ≤ *a* ≤ *F*) before the $\left( 1-q \right)G$ scroungers arrive; the rest is shared between that producer and the scroungers. This assumption, that *all* scroungers share what remains, is simplistic and doubtless not valid for large groups; numerous other assumptions could be used. However, the details of such assumptions do not influence the overall result that we are illustrating. After time *T*, this yields each producer an expected total intake of:

$$C_{p}=\lambda T\left( a+\frac{F-a}{1+\left( 1-q \right)G} \right).$$

And for each scrounger:

$$C_{s}=\lambda T\frac{qG(F-a)}{1+\left( 1-q \right)G}.$$

Under evolved conditions (and, for simplicity, ignoring differences in variance), we expect $C_{p}= C_{s}.$ This gives us the expected proportion of producers in the population:

$$q=\frac{a}{F}+\frac{1}{G} (1)$$

(Giraldeau & Beauchamp 1999; Box 1).

Note that equation 1 assumes that *q* is a function of *G* – but, in general, *G* is also a function of *q* (through ecological feedback), which is a drawback for looking at environmental change. We focus on how different mechanisms can influence outcomes following a HIREC change.

We first assume that the behavioral traits are genetic.

There are various possible HIREC changes; here we contrast just two, to show that decreasing the amount of food available may increase or decrease the proportion of producers, depending on how that change occurs:

1. The rate of food being found by producers, $\lambda$, may alter. It is immediately obvious from the equations for $C_{p}$ and $C_{s}$ that altering $\lambda$ will not affect the immediate relative success of producers and scroungers (also, similarly, altering the total foraging time, *T*).

However, if we assume that the population size, *G,* will subsequently decrease (as a result of the reduced food), then the subsequent payoffs, $C_{p}$ and $C_{s}$, will alter, as will their ratio.

Equation 1 suggests that the proportion of the population that are producers, *q,* should increase as *G* decreased, but we also know that *G* is a function of *q*; this can lead to *q* decreasing as *G* decreases. For instance, say that pre-HIREC, *G* = 10, *F* = 8, *a* = 4. Irrespective of *T* or $\lambda$, the pre-HIREC ESS is *q* = 0.6. Now suppose that HIREC reduces $\lambda$ to half its previous value. The optimal value of *q* at that point is still the actual *q* (of 0.6), but only half as much food is being generated. This is likely to subsequently reduce the population size, *G*; the extent to which *G* decreases will again depend on introducing additional assumptions. If reproductive success is proportional to the amount of food consumed (a highly simplistic assumption), one might think that *G* would halve, from 10 to 5; immediate use of equation 1 would then suggest that the optimal value of *q* increases, from 0.6 to 0.7 (i.e., a *greater* proportion of producers). However, as *G* decreases, the optimal *q* should also shift; this alters the amount of food that will be found, so in turn affects where *G* and *q* should re-balance. Assuming that *G* is proportional to $q\lambda$, then the final balance (when $\lambda$ is halved from whatever value it was previously) is *G* = 5.64, *q* = 0.58; *i.e., decreasing* $\lambda$ *results in a slightly smaller proportion of producers, q.*

1. The size (i.e., value) of food, *F*, may change. If this decreases (e.g., if humans tend to extract larger prey from the system), then although producers will do slightly worse than before on an absolute scale, they do considerably better than scroungers after HIREC. Thus, *decreasing F will result in a greater proportion of producers, q.*

Clearly, in the genetic (or genetically polymorphous) case, it will take numerous generations to settle at the new optimal balance of producers to scroungers.

In contrast with the genetic (or genetically polymorphous) case, if the species has evolved to use cues about the amount of food that is available (for instance), then it is possible that individuals may immediately respond appropriately to changes in the environment. However, if the cues are not used in the appropriate manner, which is likely in situations where HIREC takes the species into situations that were not previously experienced, the resultant behavior may be far from optimal.

In the case of behavior coming about through learning, the long-term effect will again be similar to the above cases, but rather than immediate (through inherent adaptive use of cues) or adaptation occurring over many generations, the new balance (ESS) may be struck within a lifetime. Overall, we see that the likely effects of variable environmental change over time: in the short-term, faster adapters should do better than slower adapters; i.e., reliable cue-based mechanisms do better than learners, which in turn do better than genetic fixed strategies. Note, however, that it can be better to be genetically fixed (relying on bet-hedging) than a learner if environmental switching is very fast (Stephens 1991, Botero et al. 2015).

The hawk-dove and producer-scrounger games have a negative-frequency dependent aspect: the more individuals there are playing one strategy, the more profitable the other strategy becomes. We now turn to a positively-frequency dependent case, where it is typically best for each individual to make the same choices as the other player.

The Iterated Prisoner’s Dilemma

The prisoner’s dilemma (PD; Luce & Raiffa 1957) is a simple game in which two individuals have the opportunity to cooperate with one another or ‘defect’; as shown in Table 2 (where the > symbol means `is preferred to’). The dominated best response strategy is for both players to defect, resulting in each player obtaining a payoff of *P*, even though each would obtain *R* (> *P*) if they had both cooperated.

|  |  | | **Other player** | |
| --- | --- | --- | --- | --- |
|  | | | **Cooperates** | **Defects** |
| **Focal player** | | **Cooperates** | ***R*** | ***S*** |
|  |  | **Defects** | ***T*** | ***P*** |

Table 2: The Prisoners Dilemma payoff matrix, with payoffs shown for the focal player. *T* > *R* > 0, *P* > *S*. Thus when the game will be played only once, the best strategy for each player is to defect, even though *R* > *P*.

A larger game setting involves the players interacting repeatedly, playing the same PD as a sub-game in each of multiple rounds; this is known as an iterated prisoner’s dilemma (IPD; also known as the repeated prisoner’s dilemma). If all opponents are expected to defect, then it is clearly best to defect oneself. In the case of all opponents being expected to cooperate (presumably conditionally in the future, depending on your current action), then with sufficient rounds still to be played, it is best to cooperate. If the number of rounds is known, then backward induction shows that it is best for the players to defect on each round. However, many texts have pointed out that in slightly different circumstances, such as the players potentially making errors or uncertainty over the number of rounds and being able to build trust, then cooperation can be the dominant strategy (e.g., Boyd 1989, Nowak & Sigmund 1993). We consider one such scenario here.

McNamara et al. (2004) consider an IPD in which there are a fixed number of possible rounds, but individuals interact until one of them defects (at which point the game ends). They show that sufficient variation in duration of cooperation in the population (due to extrinsic factors) can lead to the evolution of extensive levels of cooperation. This happens because, at each time step, the small risk associated with cooperating for an additional round can give worthwhile information about the other player for future decisions (following a defection, the player would choose not to cooperate further). Thus there is a balance between the possible long-term benefit of cooperating and the short-term benefit of defecting (along with the risk of cooperating). Thus, the optimal length of time to cooperate will depend on the payoff values (*R*, *S*, *T*, *P*) in each round of the IPD.

Again, we find that the outcome of IPD games following HIREC will depend strongly on the details of the decision-mechanism (evolved to meet the pre-HIREC optima):

- If the *behavior* is what has evolved (i.e., how long to conditionally cooperate for), then we can expect that behavior to be continued immediately after HIREC, even if the payoffs have altered. This would take numerous generations for behavior to re-optimise.
- If players have evolved a rule which takes into account the relative size of the payoffs and those payoffs are known ahead of each decision through cues (i.e., behaviors are the outcome of cues), then behavior can immediately shift as rewards alter with HIREC.
- If the players learn to adjust their expectations about payoff values through experience, then there are yet more options for how behaviors will alter over time following HIREC.

Supplementary references

Beauchamp, G. (2000) Learning rules for social foragers: implications for the producer-scrounger game and ideal free distribution theory. J. Theor. Biol. 207, 1-15.

Botero, C.A., Weissing, F.J., Wright, J. Rubenstein, D.R. (2015) Evolutionary tipping points in the capacity to adapt to environmental change. PNAS 112(1), 184-189.

Boyd, R. (1989) Mistakes Allow Evolutionary Stability in the Repeated Prisoner‘s Dilemma Game. J. Theor. Biol. 136, 47-56.

Giraldeau, L-A. & Beauchamp, G. (1999) Food exploitation: searching for the optimal joining policy. TREE 14(3), 102-106.

Luce, R.D. & Raiffa, H. (1957) Games and Decisions. Wiley, New York.

McNamara, J.M., Barta, Z., Houston, A.I. (2004) Variation in behaviour promotes cooperation in the Prisoner‘s Dilemma game. Nature, 428(6984), 745-748.

Nowak, M. & Sigmund, K (1993) A strategy of win-stay, lose-shift that outperforms tit-for-tat in the Prisoner's Dilemma game. Nature 364, 56-58.

Stephens, D.W. (1991) Change, regularity and value in the evolution of animal learning. Behavioral Ecology 2(1), 77-89.
